# Supplementary material for: Physicochemical and In Vitro Digestion Properties of Curcumin-Loaded Solid Lipid Nanoparticles with Different Solid Lipids and Emulsifiers
Source: Foods. 2023 May 18;12(10):2045. doi: 10.3390/foods12102045 (PMC10217647; doi:10.3390/foods12102045)
Supplement: Supplementary file 1 [file foods-12-02045-s001.zip › foods-2365466-supplementary.pdf]

Figure S1 Visual appearance of Cur-SLN dispersions under various pH values (A) and ionic strength (B) after 4 weeks storage at 4 °C.

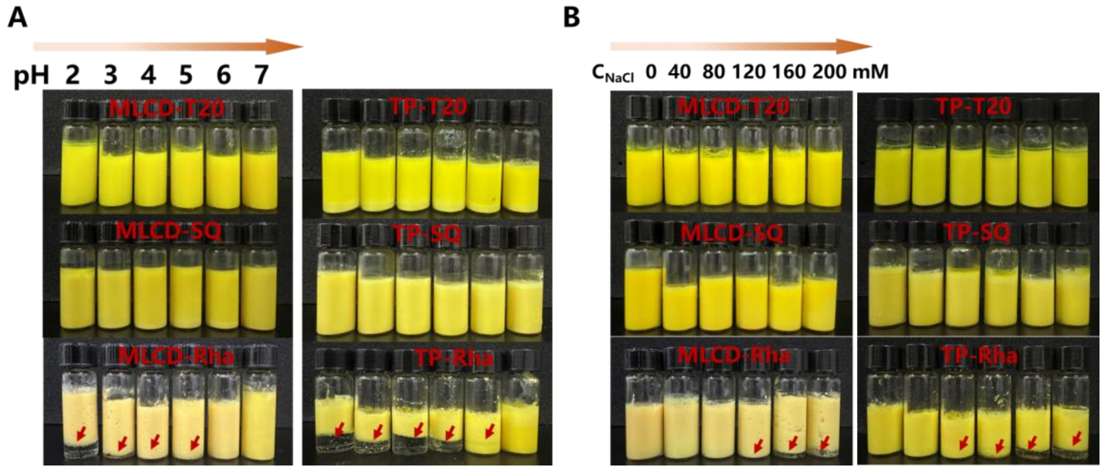

Table S1. Melting peak temperatures, crystallization onset temperatures and corresponding enthalpies of the bulk fat and Cur-SLNs.

Samples	DSC heating curve		DSC cooling curve	
	T_{peak} (°C)	Enthalpy (J/g)	T_{oneset} (°C)	Enthalpy (J/g)
Bulk MLCD	1: 51.75±0.05	1: –13.47±0.05	60.92±0.11	1: 49.54±0.15
	2: 57.27±0.12	2: –8.59±0.18		2: 7.96±0.03
	3: 68.26±0.09	3: –53.66±0.07		3: 2.20±0.02
MLCD-T20	1: 52.66±0.03	1: –1.09±0.12	36.97±0.03	1: 5.99±0.01
	2: 59.08±0.14	2: –0.02±0.03		
MLCD-SQ	1: 50.83±0.05	1: –0.91±0.09	41.05±0.06	1: 1.74±0.01
	2: 65.51±0.08	2: –0.37±0.01		
MLCD-Rha	1: 49.92±0.10	1: –0.01±0.00	44.59±0.02	1: 0.19±0.01
	2: 60.92±0.07	2: –0.34±0.02		2: 1.75±0.03
Bulk TP	1: 46.25±0.17	1: –125.96±0.48	41.67±0.09	1: 127.17±0.21
	2: 50.81±0.02	2: –24.73±0.09		
	3: 66.42±0.04	3: –131.59±0.17		
TP-T20	1: 62.75±0.10	1: –10.08±0.03	22.92±0.12	1: 4.99±0.04
TP-SQ	1: 45.33±0.01	1: –7.00±0.06	31.49±0.07	1: 3.24±0.06
	2: 65.51±0.05	2: –7.13±0.05		
TP-Rha	1: 44.42±0.02	1: –2.16±0.01	31.88±0.04	1: 0.64±0.02
	2: 55.41±0.21	2: –0.49±0.03		2: 0.55±0.07
	3: 63.67±0.13	3: –0.62±0.07		3: 0.85±0.02

Table S2. The proportion of different crystal form of SLNs after storage for 0 day and 4 weeks.

Samples	0 day			4 weeks		
	α	β	β′	α	β	β′
MLCD-T20	35.15±0.02	37.90±0.14	26.94±0.05	21.78±0.03	31.71±0.23	45.51±0.18
MLCD-SQ	32.72±0.10	47.35±0.08	19.93±0.12	34.80±0.11	25.37±0.10	39.83±0.07
MLCD-Rha	51.38±0.04	36.78±0.21	11.84±0.04	56.25±0.18	43.75±0.08	-
TP-T20	10.35±0.06	39.30±0.06	50.35±0.14	6.12±0.04	47.03±0.05	46.85±0.14
TP-SQ	100±0.00	-	-	81.94±0.09	6.78±0.04	11.28±0.03
TP-Rha	70.61±0.08	11.36±0.17	18.06±0.11	35.94±0.05	32.17±0.13	31.89±0.16

Table S3. Models analyzing the fraction of Cur released (Q) from free Cur and Cur-SLN during simulated gastrointestinal digestions.

Sample	Zero order model		First order model		Higuchi model		Ritger-Peppas model	
	Equation	R^2	Equation	R^2	Equation	R^2	Equation	R^2
Free Cur	$Q = 0.2922t + 28.1105$	0.9831	$Q = 86.1825(1- e^{-0.0137t})$	0.9493	$Q = 5.4702t^{1/2} + 5.0706$	0.9879	$Q = 10.2481t^{0.3897}$	0.9980
MLCD-T20	$Q = 0.2319t + 6.8481$	0.9528	$Q = 136.2806(1- e^{-0.0027t})$	0.9525	$Q = 4.2869t^{1/2} - 7.3447$	0.9125	$Q = 0.6372t^{0.8368}$	0.9344
MLCD-SQ	$Q = 0.1832t + 6.4436$	0.9810	$Q = 103.0443(1- e^{-0.0023t})$	0.9454	$Q = 3.2935t^{1/2} - 5.6494$	0.9247	$Q = 0.8186t^{0.7472}$	0.9711
MLCD-Rha	$Q = 0.1726t + 5.2040$	0.9819	$Q = 96.19826(1- e^{-0.0027t})$	0.9729	$Q = 3.1064t^{1/2} - 5.7782$	0.9316	$Q = 0.5900t^{0.7955}$	0.9818
TD-T20	$Q = 0.2583t + 8.6962$	0.9918	$Q = 112.0279(1- e^{-0.0036t})$	0.9704	$Q = 4.4575t^{1/2} - 7.3483$	0.9405	$Q = 1.2307t^{0.7280}$	0.9904
TP-T20	$Q = 0.2184t + 6.2948$	0.9629	$Q = 119.7439(1- e^{-0.0028t})$	0.9736	$Q = 3.9276t^{1/2} - 6.7493$	0.9370	$Q = 0.6898t^{0.80768}$	0.9498
TS-T20	$Q = 0.2086t + 6.4878$	0.9831	$Q = 132.9485(1- e^{-0.0023t})$	0.9542	$Q = 3.7219t^{1/2} - 6.9894$	0.9208	$Q = 0.9759t^{0.7341}$	0.9674
